# Items

**Published:** 1881-06

**Authors:** 


DR. FERDINAND COLETTI, Professor of Therapeutics in the University of Padua, died recently. Dr. Colletti was the first of the most zealous apostles of cremation in Italy, and he desired, in his will, that his corpse should be transmitted to Milan to be cremated there. His wish was carried out, and the body was burned in the presence of a large assemblage of citizens and of his colleagues, and of political and scientific authorities. Many orations were pronounced in his honor. The ashes were enclosed in a beautiful glass urn, and presented to his family at the expense of the Italian Cremation Society.

THE thirty-second annual session of the Medical Association of Georgia, was held in Thomasville, on April 20 and 21, 1881. The following are the officers for the ensuing year: President, William F. Holt, Macon; First Vice President, Eugene Foster, Augusta; Second Vice President, T. M. McIntosh, Thomasville; Secretary, A. Sibley Campbell, Augusta; Treasurer, K. P. Moore, Forsyth. The next session will be held in Atlanta on the third Wednesday in April (19th) 1882. Communications should be addressed to A. Sibley Campbell, Secretary.

ANATOMICAL PLATES.—Messrs. G. P. Putnam & Sons desire to state that, through a clerical error, the name of the late Prof. GRANVILLE SHARP PATTISON, as translator and editor of the edition of MASSE'S ANATOMICAL PLATES, issued in 1845, has been omitted from the title page of their present edition. They would also explain that Prof. RANNEY'S labor as editor embraced such alterations of the plates and text as was required to bring these fully up to date, together with the preparation of important new material in the way of diagrams and descriptive text.

*Please paste over preface of "Anatomical Plates" of Masse, and oblige*

*A. L. RANNEY, M.D.*

A larger portion of this preface should be accredited to the late Prof. PATTISON, a former editor of these plates, to whom due credit has been omitted by an oversight on the part of the present editor.

